# Regulating Th17/Treg Balance Contributes to the Therapeutic Effect of Ziyuglycoside I on Collagen-Induced Arthritis

**DOI:** 10.3390/ijms232416105

**Published:** 2022-12-17

**Authors:** Manman Wang, Tiantian Su, Hanfei Sun, Huijuan Cheng, Chunru Jiang, Paipai Guo, Zhenduo Zhu, Ruhong Fang, Feng He, Mingli Ge, Qiuyun Guan, Wei Wei, Qingtong Wang

**Affiliations:** Institute of Clinical Pharmacology, Anhui Medical University, Key Laboratory of Anti-Inflammatory and Immune Medicine, Ministry of Education, Anhui Collaborative Innovation Center of Anti-Inflammatory and Immune Medicine, Hefei 230032, China

**Keywords:** rheumatoid arthritis, Th17/Treg, Ziyuglycoside I, mTOR, RORγt, Foxp3

## Abstract

To investigate the therapeutic effect and primary pharmacological mechanism of Ziyuglycoside I (Ziyu I) on collagen-induced arthritis (CIA) mice. CIA mice were treated with 5, 10, or 20 mg/kg of Ziyu I or 2 mg/kg of methotrexate (MTX), and clinical manifestations, as well as pathological changes, were observed. T cell viability and subset type were determined, and serum levels of transforming growth factor-beta (TGF-β) and interleukin-17 (IL-17) were detected. The mRNA expression of retinoid-related orphan receptor-γt (RORγt) and transcription factor forkhead box protein 3 (Foxp3) in mouse spleen lymphocytes was ascertained by the real-time reverse transcriptase-polymerase chain reaction (RT-qPCR). Molecular docking was used to detect whether there was a molecular interaction between Ziyu I and protein kinase B (Akt). The activation of mechanistic target of rapamycin (mTOR) in T cells was verified by Western blotting or immunofluorescence. Ziyu I treatment effectively alleviated arthritis symptoms of CIA mice, including body weight, global score, arthritis index, and a number of swollen joints. Similarly, pathological changes of joints and spleens in arthritic mice were improved. The thymic index, T cell activity, and RORγt production of Ziyu I-treated mice were significantly reduced. Notably, through molecular docking, western blotting, and immunofluorescence data analysis, it was found that Ziyu I could interact directly with Akt to reduce downstream mTOR activation and inhibit helper T cell 17 (Th17) differentiation, thereby regulating Th17/regulatory T cell (Treg) balance and improving arthritis symptoms. Ziyu I effectively improves arthritic symptoms in CIA mice by inhibiting mTOR activation, thereby affecting Th17 differentiation and regulating Th17/Treg balance.

## 1. Introduction

Rheumatoid arthritis (RA) commonly having a pattern of first manifesting clinically as chronic inflammation in the synovial membrane, followed by cartilage and bone erosion [1]. Although the precise pathogenesis of RA is unclear, both T cell and B cell activation is involved in the pathogenesis of RA [2]. Due to the continuous infiltration and activation of lymphocytes, joint synovial tissue suffers from abnormal proliferation, causing joint deformities and dysfunction. Helper T cell 17 (Th17) and regulatory T cell (Treg) are developed by the activation of initial CD4+ T cells stimulated by foreign antigens. It has been demonstrated that an imbalance in Th17/Treg cells has an important role in joint inflammation and destruction [3].

At present, many anti-rheumatic medications have serious adverse reactions, which makes the development of new drugs to fight against RA safely an important focus of current pharmacological exploration. Many studies have exhibited that naturopathic remedies have significant effects on the function of the immune system for the prevention and treatment of RA while also being less toxic and causing fewer adverse side effects [4,5]. *Sanguisorba* is a well-established herbal plant with medicinal uses. *Sanguisorba* is rich in chemical components containing phenols, flavonoids, triterpenoids, and other small molecule compounds. Ziyuglycoside I (Ziyu I) and Ziyu II in *Sanguisorba* have good anti-inflammatory and anticancer effects [6]. For instance, Ziyu I exhibits a diverse array of promising properties in preliminary reports, such as anti-inflammatory [7,8,9], antiedematous, anti-tumor, antimutagenic, venotonic, expectorant, and broncholytic activities [10,11]. What remains to be explored fully is the anti-rheumatism effects of Ziyu I. 

To further investigate how Ziyu I impacts RA, collagen-induced arthritis (CIA) mice were treated over 4 weeks with varying dosages of Ziyu I and compared with CIA mice treated with the positive control, methotrexate (MTX) or treated with vehicle as the negative control. The mice demonstrated an improvement in joint inflammation and a down-regulation of the Th17 cell ratio after Ziyu I treatment, suggesting Ziyu I is a promising anti-RA drug.

## 2. Results

### 2.1. Ziyu I Ameliorates Arthritis Manifestations of CIA Mice

**Figure 1:**
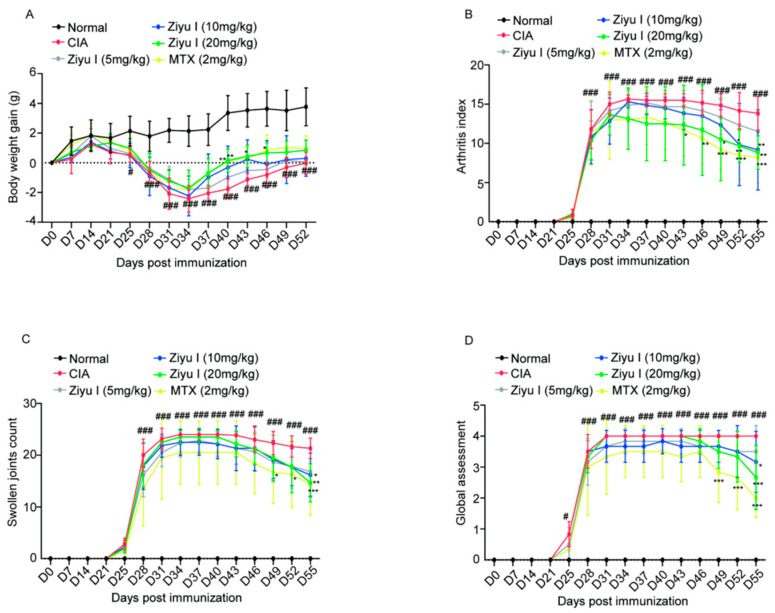
Ziyu I treatment effectively improves the manifestations of mice with CIA. (**A**). Body weight gain (g) (**B**). Arthritis index (**C**). Swollen joint count (**D**). Global scoring. The data were expressed as mean ± SD, n = 5~6. # *p* < 0.05, ### *p* < 0.001 vs. Normal. * *p* < 0.05, ** *p* < 0.01, *** *p* < 0.001 vs. CIA.

The body weight of CIA mice decreased significantly on day 24 compared with that of control mice after the first injection of chicken collagen type II (CCII) emulsion and gradually recovered until day 34, but the body weight of CIA mice was significantly lower than that of the normal control group. 20 mg/kg Ziyu I significantly promoted body weight increase in CIA mice at 40 and 43 days, while MTX significantly promoted body weight gain in CIA mice at 46 days (Figure 1A). After injection of the booster, some mice experienced signs of arthritis, such as paw swelling, as early as day 25, while all immunized mice developed joint swelling by day 28. On day 25, some CIA model mice not only developed joint swelling but also had nodules of connective tissue hyperplasia in the nose, tail, and/or ear. By day 55, CIA mice treated with the vehicle still had severe joint inflammation. From day 49, the arthritis index of CIA mice was significantly alleviated in the group treated with 20 mg/kg Ziyu I, while earlier pharmacological responses began to appear in MTX treatment from day 43 (Figure 1B). Consistent with swollen joints, mice immunized with CCII had a significantly higher arthritis index, which was significantly ameliorated not only by MTX but also by 20 mg/kg Ziyu I and 10 mg/kg Ziyu I (Figure 1C). From day 28 onwards, immunized mice scored 2–4 on the global assessment. After MTX treatment, the overall score of mice decreased from day 49, while Ziyu I administration of 10 mg/kg and 20 mg/kg improved the overall performance of CIA mice to varying degrees (Figure 1D). The data indicate that the Ziyu I treatment was able to effectively alleviate the inflammatory response of CIA mice, although the onset time was later compared with MTX. Therefore, we speculated that Ziyu I may be a candidate natural compound for the treatment of arthritis.

### 2.2. Ziyu I Improves the Pathologic Change of Joints and Spleens of CIA Mice

**Figure 2:**
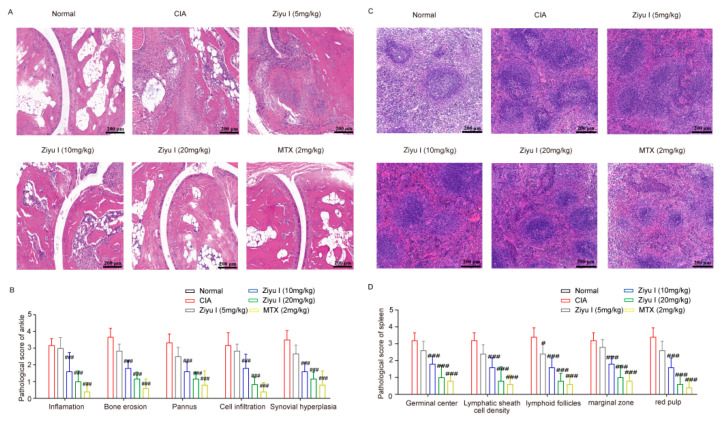
Effects of different doses of Ziyu I on the histopathology of ankles and spleens of CIA mice. (**A**). Representative pathological images of ankles. (**B**). Analysis of pathological ankle scores. (**C**). Representative pathological images of the spleen. (**D**). Analysis of pathological spleen scores. The data were expressed as mean ± SD, n = 5. # *p* < 0.05, ### *p* < 0.001 vs. CIA.

When histological evidence was evaluated, it was seen that the joints of CIA mice have severe articular cartilage destruction, inflammatory synovial infiltration, and synovial cell proliferation, as well as bone and synovium erosion. 10 mg/kg or 20 mg/kg Ziyu I or MTX treatment significantly reduced the articular cartilage damage, angiogenesis, and synovial hyperplasia of CIA mice, while 5 mg/kg Ziyu I had no significant action in the improvement of joint pathology score in CIA mice (Figure 2A,B). The normal splenic structure consists of two major functional areas: the hematopoietic red pulp and the lymphatic white pulp. The white pulp consists of the lymphatic follicles and the lymphatic sheath around the arterioles. In CIA mice, the number of germinal centers (GCs) and cell density around the lymphatic sheaths were notably increased, and the lymphoid follicles, marginal zone, and red pulp had substantial cell proliferation. It is worth noting that 5 mg/kg Ziyu I treatment did reduce lymphoid follicles in the spleen of CIA mice. Furthermore, 10 mg/kg, 20 mg/kg Ziyu I, or MTX treatment significantly reduced the frequency of GCs, cell density around the lymphatic sheath, lymphoid follicle size and marginal zone, and red pulp congestion in varying degrees (Figure 2C,D). When taken together, Ziyu I treatment significantly inhibited the pathological changes of inflamed joints, which was in line with the diagnostic signs of Ziyu I treatment. The data also depicts that Ziyu I was able to significantly improve the spleen pathology of CIA mice.

### 2.3. Ziyu I Restores the Th17/Treg Balance of CIA Mice

**Figure 3:**
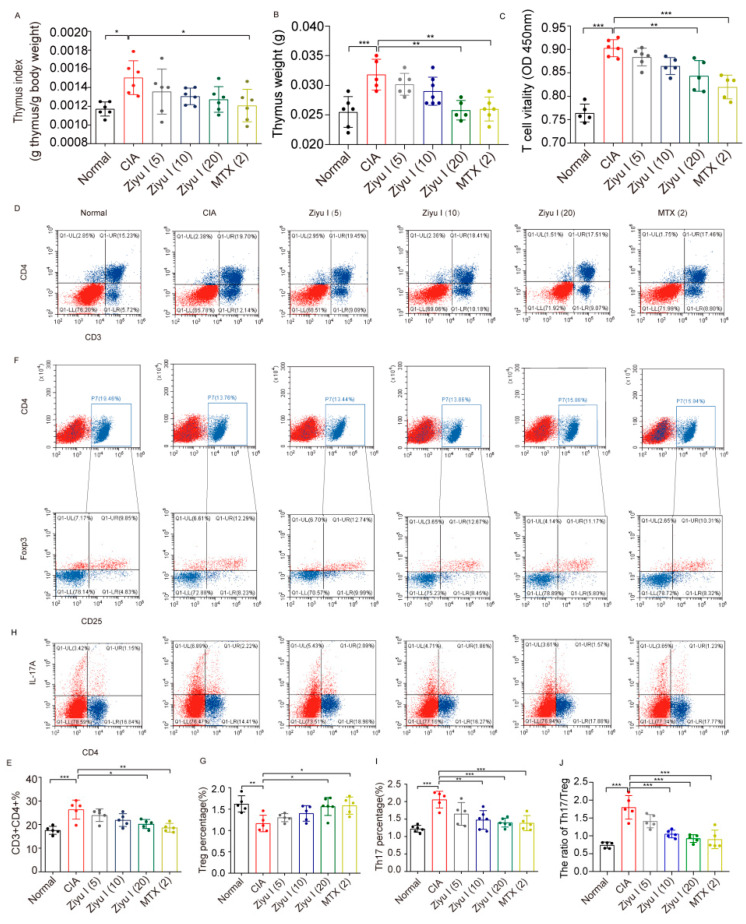
Ziyu I treatment reduces thymus pathology and the proportion of Th17 cells in CIA mice. (**A**). The thymus index (g thymus/g body weight) of treated CIA mice was calculated (**B**). Thymus weight (g) (**C**). T cells vitality was tested by CCK-8 (**D**). Representative flow cytometry scatter plot of CD3+CD4+ Th cells (**E**). The percentage of Th cells was analyzed (**F**). Representative flow cytometry scatter plot of CD4+CD25+Foxp3+ Treg cells (**G**). The percentage of Treg cells was analyzed (**H**). Representative flow cytometry scatter plot of Th17 cells (**I**). The percentage of Th17 cells was analyzed (**J**). The ratio of Th17/Treg cells was analyzed. * *p* < 0.05, ** *p* < 0.01, *** *p* < 0.001 vs. CIA. (n = 5–6, mean ± SD).

CIA mice had an increased thymus index in comparison to control mice. 20 mg/kg Ziyu I tended to decrease the thymus index (Figure 3A). Thymus weight and thymus index had the same trend (Figure 3B). Cell Counting Kit-8 (CCK-8) testing was performed on lymphocytes collected from the thymus of mice in each treatment group to determine the activity of T cells after stimulation with concanavalin A (ConA). T cell activity in CIA mice was significantly higher than found in normal splenic T cells, which was highly inhibited by 20 mg/kg Ziyu I or MTX treatment. This suggests that Ziyu I prevents T cell overreaction in arthritic mice (Figure 3C). CIA mice had a significant increase in the total number of CD3+CD4+ Th cells compared with the normal group, while the total Th cells in 20 mg/kg Ziyu I treated mice were decreased by significant degrees (Figure 3D,E).

CD4+CD25+ transcription factor forkhead box protein 3 + (Foxp3+) cells were labeled to analyze the proportions of Treg cells in the splenic cell. The proportion of Treg cells in the spleen of CIA mice treated with Ziyu I was significantly increased, which was statistically significant. 20 mg/kg Ziyu I or MTX significantly increased the proportion of Treg cells (Figure 3F,G). Th17 cells play an important pathogenic role in RA and mediate autoimmune responses to local inflammation and joint destruction [12]. Therefore, flow cytometry was used to detect the percentage of Th17 in each group. The results depict that Th17 cells in the CIA spleen increased significantly, which was statistically significant. 10 mg/kg Ziyu I, 20 mg/kg Ziyu I or MTX could significantly inhibit the proportion of Th17 cells (Figure 3H,I). Ziyu I was able to improve the balance of Th17/Treg to varying degrees (Figure 3J). These data further confirm that cellular immunity homeostasis can be largely restored upon Ziyu I treatment because Ziyu I is able to improve the Th17/Treg imbalance by increasing the percentages of Treg cells and reducing the percentages of Th17 cells.

### 2.4. Ziyu I Restores Serum Levels of Cytokines Transforming Growth Factor-beta (TGF-β) and Interleukin-17 (IL-17) as Well as the Gene Expression of Retinoid-Related Orphan Receptor-γt (RORγt) and Foxp3

**Figure 4:**
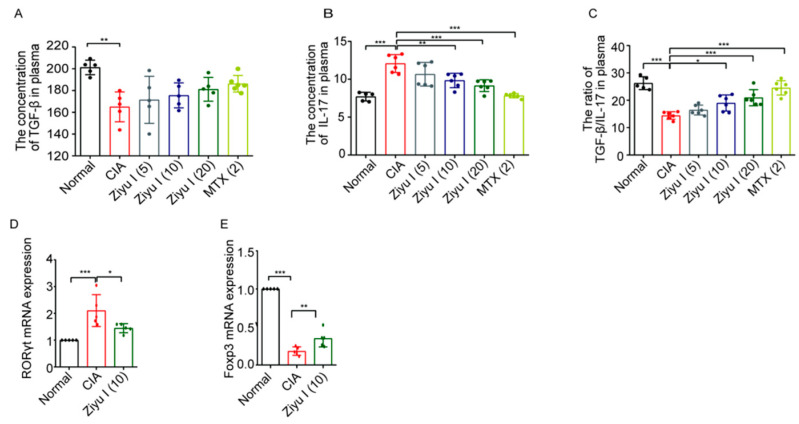
Effects of different doses of Ziyu I on splenic T cells of CIA mice. (**A**). Plasma TGF-β level (pg/mL) (**B**). Plasma IL-17 level (pg/mL). The concentration was calculated according to the absorbance at 450 nm. (**C**). Plasma TGF-β/IL-17 level (**D**). The mRNA expression of RORγt in splenic lymphocytes from the indicated group of mice was determined by RT-qPCR (**E**). The mRNA expression fold change of Foxp3 in splenic lymphocytes from the indicated group of mice was determined by RT-qPCR. * *p* < 0.05, ** *p* < 0.01, *** *p* < 0.001 vs. CIA. (n = 5–6, mean ± SD).

On day 55, after the mice were anesthetized, blood serum was collected by centrifugation after standing at room temperature for 2 h. The levels of TGF-β and IL-17 in the serum were detected by enzyme-linked immunosorbent assay (ELISA). TGF-β is a pleiotropic cytokine that has important regulatory effects on cell growth, differentiation, and immune function and can inhibit the proliferation of immunocompetent cells [13]. IL-17 is one of the most important pro-inflammatory cytokines in RA pathogenesis. Compared with normal mice, the level of IL-17 was significantly increased, and the level of TGF-β was significantly decreased in CIA mice (Figure 4A–C). 10 mg/kg Ziyu I, 20 mg/kg Ziyu I, or MTX treatment significantly decreased IL-17 serum concentration and similarly reduced the IL-17/TGF-β ratio, suggesting that Ziyu I has pivotal effects on cell signaling cascades that are important for T-cell type switching.

Splenic lymphocytes of the indicated group of mice were isolated, and the mRNA expression levels of RORγt and Foxp3 in lymphocytes were detected by the real-time reverse transcriptase-polymerase chain reaction (RT-qPCR). The expression level of RORγt in the 10 mg/kg Ziyu treated group was significantly lower than that of the CIA model group, while the expression level of Foxp3 in the treatment group was significantly higher than that of the CIA model group, suggesting that Ziyu I was able to increase Foxp3 and decrease RORγt mRNA expression level (Figure 4D,E). These data further confirm that Ziyu I is able to reduce the proportion of pro-inflammatory cells in CIA mice by restoring immune homeostasis and improving joint symptoms.

### 2.5. In Silico Docking Analysis Indicates Ziyu I Interacts with Akt1

**Figure 5:**
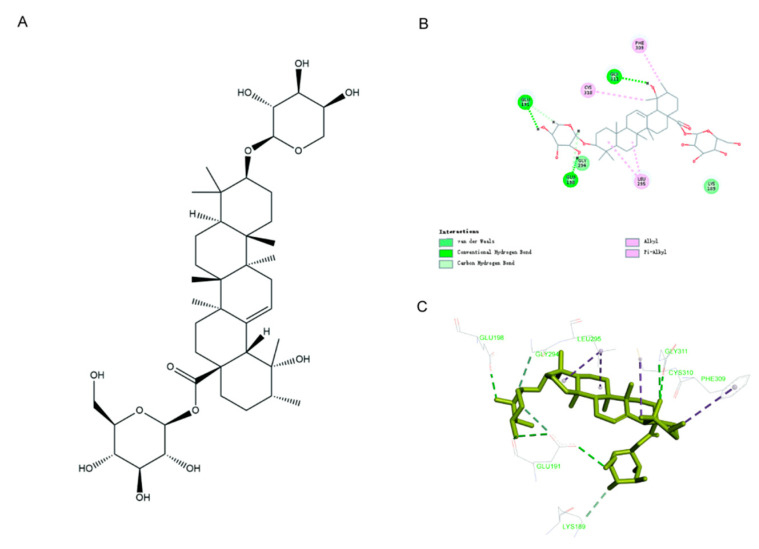
Molecular docking results for Ziyu I docking with Akt1. (**A**) Molecular structure of Ziyu I (**B**). A planar view of the docking results of Ziyu I and Akt1. The dashed line represents the existing interaction force between the ligand and receptor. (**C**). Three-dimensional diagram of the docking results of Ziyu I and Akt1. Yellow represents the Ziyu I, and green marks the amino acid residues. The dotted line represents the existing interaction force between the ligand and the receptor.

The above data indicated that Ziyu I was able to inhibit the differentiation of Th17, and it is known that many signaling pathways can be involved in Th17 differentiation, such as Notch, toll-like receptor 7 (TLR7), or (phosphoinositide 3-kinase) PI3K/mechanistic target of rapamycin (mTOR) signaling [14,15,16], but which signaling mechanism would be the target of Ziyu I is not clear at present. Subsequently, molecular docking analysis was performed to screen the potential target of Ziyu I, which has a molecular weight of 766.95 kDa and a formula of C_41_H_66_O_13_ (Figure 5A). The amino acid residues of protein kinase B 1 (Akt1) were found from NCBI, and the 3D protein structure of Akt1 was established by using the homology modeling function of discovery studio (DS) software. As a result, Akt1 portrayed a binding affinity with Ziyu I, with interaction occurring by hydrogen bonds, van der Waals forces, and non-covalent bonds. This suggests that Akt1 can directly interact with Ziyu I (Figure 5B). A three-dimensional diagram of the docking results of Ziyu I and Akt1 is also presented (Figure 5C). The data indicate that Ziyu I may affect the differentiation of Th17 cells by regulating Akt1 activity and downstream mTOR signaling. However, this prediction needs to be verified with further experimentation.

### 2.6. Ziyu I Reduces the Activation of mTOR in T Cells of CIA Mice

**Figure 6:**
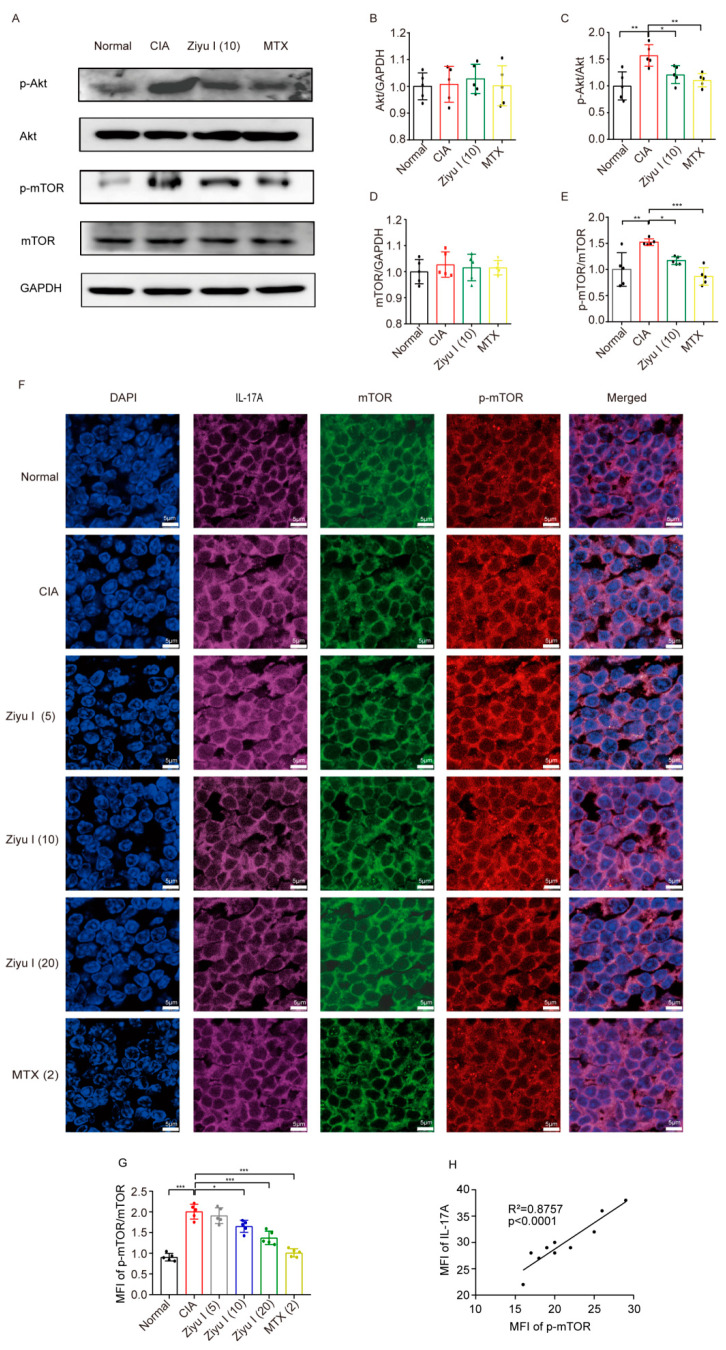
Ziyu I treatment reduces the activation of mTOR in T cells of CIA mouse. (**A**). The relative expression of Akt, mTOR, and p-Akt, p-mTOR in splenic T cells from normal mice, CIA mice, and 10 mg/kg Ziyu I treatment group of mice (**B**). The analysis of Akt/GAPDH expression (**C**). The analysis of p-Akt/Akt expression (**D**). The analysis of mTOR/GAPDH expression (**E**). The analysis of p-mTOR/mTOR expression (**F**). Representative immunofluorescence images of mTOR, p-mTOR, and IL-17A labeling in spleen tissue of treated mice (**G**). The mean fluorescence intensity of p-mTOR/mTOR in the spleens of mice was analyzed by immunofluorescence (**H**). Correlation analysis between MFI of p-mTOR and MFI of IL-17A in the spleen tissue of CIA mice. The data are presented as mean ± SD, n = 5. * *p* < 0.05, ** *p* < 0.01, *** *p* < 0.001 vs. CIA.

In order to test this hypothesis, the activation of mTOR in T cells from treated mice was measured. Levels of Akt, phospho-Akt (p-Akt), mTOR, and phospho-mTOR (p-mTOR) were analyzed by Western blotting. Compared with normal mice, the expression of p-mTOR and p-Akt in T cells from CIA mice was significantly increased. However, the activation of mTOR or Akt in splenic T cells of CIA mice was significantly reduced by 10 mg/kg of Ziyu I treatment (Figure 6A–E). Furthermore, the in-situ expression of both p-mTOR and mTOR in IL-17A-positive cells of the spleen from each treated group was detected by immunofluorescence. The results indicated that 10 mg/kg, 20 mg/kg Ziyu I or MTX therapy significantly reduced the phosphorylation rate of mTOR. Moreover, the fluorescence intensity of p-mTOR and IL-17A was significantly positively correlated (Figure 6F–H).

## 3. Discussion

In this study, it has been revealed that Ziyu I is able to effectively reduce clinical parameters, joint swelling, and circulating pro-inflammatory cytokines and improve joint and pathological spleen scores in CIA mice. MTX is currently the first-line drug for the treatment of RA, so in this work, MTX was set as the positive control drug. Although, as portrayed in the data, Ziyu I has relatively lower efficacy than MTX in treating CIA mice, the global assessment and pathological examinations definitely confirm that Ziyu I has a specific anti-arthritis property. The mild action of Ziyu I in CIA treatment may be due to its low bioavailability because of its high-fat solubility [17]. Therefore, the bioavailability of Ziyu I can be increased by changing its structure or formulating targeted drugs for specific tissue sites. Implementation of a similar docking analysis as performed in this study with Ziyu I derivative compounds will allow for culling of compounds that perform poorly during the in silico studies, henceforth allowing the wet bench experiments to have a more focused approach.

In the present study, T cell subsets in Ziyu I- or vehicle-treated CIA mice were measured to explore the potential pharmacological mechanism of Ziyu I in CIA treatment. The proportion of Th17 cells in spleen T lymphocytes of CIA mice was significantly higher than that of control mice. On the contrary, compared with normal mice, the pool of splenic Treg cells in CIA mice was slightly reduced, leading to an imbalance of Th17/Treg, which is an important step in the pathogenesis of RA [18,19]. Compared with CIA mice treated by vehicle, Ziyu I administration effectively reduces thymus index, T cell viability, plasma IL-17 concentration, and most importantly, the frequency of the Th17 cell subset. The data suggest that the balance of Th17/Treg may be a vital target of Ziyu I in alleviating experimental arthritis. 

Th17 and Treg differentiation are controlled by various signals individually [14,15]. However, the Akt-mTOR signaling pathway has been reported to modulate the metabolic profile of T cells and thus has a dual effect on both Th17 and Treg formation. Studies have found that mTOR is a key regulator of T cell glycolysis and metabolism, and its loss leads to the reduction of Th17 development but promotes the differentiation of Treg cells [20,21]. Molecular docking simulations reveal a direct interaction of Ziyu I with Akt1 by hydrogen bonds, van der Waals forces, and non-covalent bonds, and furthermore, the data presented here also displays that Ziyu I substantially reduces the elevated activation of Akt1 and mTOR in T cells from CIA mice with no influence on the basal expression level of both proteins. How Ziyu I inhibit the activation of Akt remains to be addressed.

The main active metabolite of Ziyu I in the body is Ziyu II, which has also been observed to induce cell autophagy by inhibiting the Akt/mTOR pathway [17,22]. Ziyu I and Ziyu II have similar structures and share the same parent nucleus [17]. As evidenced in the molecular docking data, the interacting sites of Ziyu I to Akt1 are primarily located in the parent nucleus, conveying that this structure is a promising candidate for Akt1 inhibitor development.

## 4. Materials and Methods

### 4.1. Induction and Treatment of Collagen-Induced Arthritis in Mice

8–10-week-old male DBA/1 mice (Shanghai SLAC Laboratory Animal Co., Ltd., Shanghai, China) housed in a pathogen-free laboratory at the Institute of Clinical Pharmacology, Anhui Medical University. All protocols of these experiments have been approved by the Ethics Committee of the Institute of Clinical Pharmacology, Anhui Medical University (Approval ID: PZ-2020-045). CIA was achieved in mice, as previously published [23]. In short, mice were injected at the base of the tail on day 0 and day 21 with an emulsification containing equal parts 1) CCII (2 mg/mL o immunization grade CCII (Catalog #:20011, Chondrex, Inc., Woodinville, WA, USA) dissolved in 0.05 M acetic acid, and 2) Complete Freund’s Adjuvant (CFA) containing 2 mg/mL heat-killed *Mycobacterium tuberculosis* (Catalog #:7009, Chondrex, Inc., Woodinville, WA, USA) [24].

95% of mice placed on the CIA injection protocol developed arthritis. CIA mice were randomly divided into treatment groups of the vehicle; 5 mg/kg, 10 mg/kg, or 20 mg/kg of Ziyu I (Catalog #: B20294, HPLC ≥ 98%, Shanghai yuanye Bio-Technology Co., Ltd., Shanghai, China); or 2 mg/kg of MTX. Treatment was started on D28 with Ziyu I once daily and MTX once every 3 days for 4 weeks. Global assessments were performed every 7 days before booster immunization and every 3 days thereafter. The change in body weight was calculated by subtracting the body weight of each mouse on day 0. The arthritis severity of each paw was evaluated by using a macroscopic scoring system ranging from 0 to 4 (0, paws with no swelling or focal redness; 1, mild but definite redness and swelling of the ankle or wrist or apparent redness and swelling limited to individual digits, regardless of the number of affected digits; 2, moderate redness and swelling of the ankle or wrist; 3, severe redness and swelling of the entire paw, including digits; and 4, maximally inflamed limbs with the involvement of multiple joints). The cumulative score for all four paws of each mouse was used as the polyarthritis index and had a maximum value of 16 [25].

### 4.2. Histopathological Examination of the Spleen and Joints

All mice were sacrificed under anesthesia on day 55. The left rear ankle joint and spleen were collected after treatment. The thymus was weighed to calculate the thymus index by comparing the thymus weight (g) to body weight (g). The ankle joints were collected from indicated mice and fixed in formalin for 24 h, then were subjected to decalcification in 10% EDTA. 4 μm slices of paraffin-embedded joints and spleens were stained for H&E were imaged with a 3D HISTECH panoramic scanner and analyzed with caseviewer software 2.4.0.119028 (3DHISTECH Ltd., Budapest, Hungary). The evaluation criteria for histology images, as well as for the grades of joints and spleens, have been previously reported [26]. The severity of joint destruction was classified from grade 0 to grade 4 for the intensity of the lining layer hyperplasia, mononuclear cell infiltration, and pannus formation. Five parameters were applied for the pathological evaluation of the spleen: the amount of red pulp, the total number of GCs, cellularity of the periarteriolar lymphoid sheath (PALS), lymphoid follicles, and marginal zone. The inflammation was ranked on a numerical scale from 0–4 (severe change) [26]. 

### 4.3. Serum Cytokine Detection with ELISA

After the mice were anesthetized, peripheral blood was collected for coagulation at room temperature for 2 h and centrifuged at 2500 rpm for 30 min at 4 °C. The supernatant was retained, and the cytokines, including IL-17 and TGF-β, were detected using ELISA kits [27]. Mouse IL-17 ELISA Kit (Catalog #: ml037866-J) and Mouse TGF-β ELISA Kit (Catalog #: ml057830-J) were products from Shanghai Enzyme-linked Biotechnology Co., Ltd., Shanghai, China, with absorbance recorded at 450 nm using a BioTek Elx×808 microplate reader (Lonza Group, Ltd., Basel, Switzerland).

### 4.4. Viability of T Lymphocytes

The viability of T lymphocytes was determined by CCK-8 assay as previously described [26]. After treatment, T cells were prepared from the thymus of mice. 1 × 10^6^ cells were seeded evenly into each well of a 96-well plate and stimulated with 5 mg/L ConA (Catalog #: FMS-FZ203, FcMACS, Nanjing, China) in a 5% CO_2_ cell incubator for 46 h. Two hours before ConA treatment was complete, 10 μL of CCK-8 solution (Catalog #: E-CK-A361, Elabscience Biotechnology, Wuhan, China) was added to each well. At the end of the treatment regimen, the absorbance at 450 nm was determined with a BioTek Elx×808 reader (Lonza Group, Ltd., Basel, Switzerland).

### 4.5. Flow Cytometry

The ratios of T lymphocyte subsets in the spleen of treated mice were detected with flow cytometry. Spleens were grounded with mortar and pestle in lymphocyte separation medium (Catalog #: DKW33-R0100, Dakewe Biotech Co., Ltd., Shenzhen, China) and then incubated with FITC Rat Anti-Mouse CD4 (Clone RM4-5, Catalog #: 553046, BD Biosciences, Franklin Lakes, NJ, USA)/APC Rat Anti-Mouse CD25 (Clone PC61, Catalog #: 561048, BD Biosciences, Franklin Lakes, NJ, USA)/PE Anti-Mo/Rt Foxp3 (Clone FJK-16a, Catalog #: 2344844, Elabscience Biotechnology, Wuhan, China)/APC Rat Anti-Mouse CD3 (Catalog #: 554832, BD Biosciences, Franklin Lakes, NJ, USA)/PE Rat Anti-Mouse IL-17A (Catalog #: 553046, BD Biosciences, Franklin Lakes, NJ, USA) and analyzed with a Cytoflex Platform (Cytoflex S, Beckman Coulter Life Sciences, Indianapolis, IN, USA). The percentage of CD4+IL-17A+ Th17 cell and CD4+CD25+Foxp3+ Treg cells in total T cells were analyzed by using CellQuest™ 2.1 software (BD, San Diego, CA, USA).

### 4.6. RT-qPCR

Total RNA was extracted from spleen cells using Trizol reagent following the manufacturer’s protocol. Complementary DNA (cDNA) was then produced with the implementation of a cDNA synthesis kit (Catalog #: 634926, Takara Bio Inc., Otsu, Shiga, Japan) referring to the instructions. The specific genes from the cDNA template were then amplified with a 7500 real-time PCR system (Applied Biosystems, Foster City, CA, USA) with the implementation of Fast SYBR Green Master Mix (Catalog #: 4385612, Thermo Fisher Scientific, Waltham, MA, USA). The specific primers for the amplified genes: RORγt, Foxp3, and GAPDH are listed in Table 1. Expression changes were calculated with the normalization of GAPDH values with the 2^−ΔΔCt^ method.

### 4.7. Molecular Docking Analysis

There is no complete three-dimensional protein structure of Akt1 in the PDB protein database. Therefore, the human amino acid sequence of Akt1 was obtained from the NCBI-Protein database (Akt1. https://www.ncbi.nlm.nih.gov/protein/, accessed on 20 November 2021), and the 3D structure was predicted with the aid of Discovery Studio 2020 software (DS 2020, Accelrys Software Inc., San Diego, CA, USA) which was informed by solved structures of component domains from the PDB _nr95 (PDB non-redundant structure database). A docking simulation of Ziyu I with Akt1 was also performed in Discovery Studio 2020. The active sites were defined in a sphere of 13.7 Å according to the important amino acid residues generated around the Akt1 binding pocket. The structure of a protein with a substrate attached was subjected to energy minimization using the CHARM force field as implemented in DS 2020. A full potential final minimization was then used to refine the substrate configuration. Based on LibDock, energy-docked conformation of the substrate was retrieved for post-docking analysis [28].

### 4.8. Western Blotting

The levels of Akt, mTOR, p-Akt, and p-mTOR in splenic T cells were ascertained by Western blotting. Cells were lysed with an ultrasonic homogenizer at 4 °C in a buffer containing phenylmethylsulfonyl fluoride (PMSF) and phosphatase inhibitor. The soluble protein was collected after pelleting the insoluble fraction at 12,000 rpm for 30 min at 4 °C. The resultant solution concentrations were quantified with a Pierce BCA Protein Assay Kit (Catalog #: 23227, Thermo Fisher Scientific, Waltham, MA, USA). Proteins were separated by 10% sodium dodecyl sulfate (SDS) polyacrylamide gel electrophoresis and then transferred to the polyvinylidene fluoride (PVDF) membrane. The membrane was blocked in TBS containing 0.05% Tween 20 (TBST) and 5% nonfat milk at 37 °C for 2 h, followed by the incubation of primary antibodies against mTOR (1:200, Catalog #: 55306F, ABMART, Shanghai China), p-mTOR (1:500, Catalog #: 293133, Santa Cruz Biotechnology, Santa Cruz, CA, USA), Akt1/2/3 mAb (1:200, Catalog #: 55561F, ABMART, Shanghai China), p-Akt (Ser473) mAb (1:200, Catalog #: T56569F, ABMART, Shanghai, China), and/or GAPDH (1:5000, Catalog #: AF0911, Affinity Biosciences, Changzhou, China) overnight at 4 °C. After washing with TBST. The membrane was placed in appropriate secondary antibody (1:10,000, Goat Anti-Rabbit IgG (H+L) HRP, Catalog #: S0001, Goat Anti-Mouse IgG (H+L) HRP, Catalog #: S0002, Affinity Biosciences, Changzhou, China) at 37 °C for 2 h. ECL Western Blotting Substrate (Catalog #: 32106, Thermo Fisher Scientific, Waltham, MA, USA) and an Image Quant LAS 500 imager (GE Healthcare Systems, Chicago, IL, USA) were implemented for band detection, with proteins being normalized to GAPDH signal and semi-quantified with ImageJ (version 1.42q, NIH) [29].

### 4.9. Immunofluorescence

In situ expression of p-mTOR/mTOR in T cells of treated mice was detected by immunofluorescence. Mouse spleen tissues were deparaffinized, permeabilized in 0.1% Triton X-100 in phosphate buffered saline (PBS) for 30 min, and blocked in PBS with 1% bovine serum albumin (BSA) for one hour. Samples were then incubated with a mixture of rabbit mAb Anti-mTOR (1:100, Catalog #: 55306F, ABMART, Shanghai China) and mouse mAb Anti-p-mTOR (1:100, Catalog #: 293133, Santa Cruz Biotechnology, Santa Cruz, CA, USA) and PE Anti-Mouse IL-17A antibody (1:100, Catalog #: 1995433, BD Biosciences, Franklin Lakes, NJ, USA) overnight at 4 °C. After rinsing with PBS. The samples were incubated with a solution containing the fluorescent secondary antibodies Alexa Fluor 647 AffiniPure Goat Anti-Mouse immunoglobulin (Ig) G (Catalog #: 115-605-205, Jackson ImmunoResearch Inc., West Grove, PA, USA) or Alexa Fluor 594 AffiniPure Goat Anti-Rabbit IgG (Catalog #: 115-585-003, Jackson ImmunoResearch Inc., West Grove, PA, USA) for 2 h. After washing with PBS, DAPI was applied for 10 min to stain the nuclei, and the slides were sealed with a drop of anti-fluorescence quenching mounting solution. A Leica TCS SP8 laser scanning confocal microscope and ImageJ software were employed to capture and quantify the resulting images.

### 4.10. Statistical Analysis

Data were analyzed in GraphPad Prism software (version 6, GraphPad Software, Inc., San Diego, CA, USA) and expressed as mean ± standard deviation (SD). One-way analysis of variance (ANOVA) was used to determine significant differences between three or more groups. Two-way ANOVA was used to determine significant differences between three or more groups where time was also a variable. *p* < 0.05 was considered significant.

## 5. Conclusions

**Figure 7:**
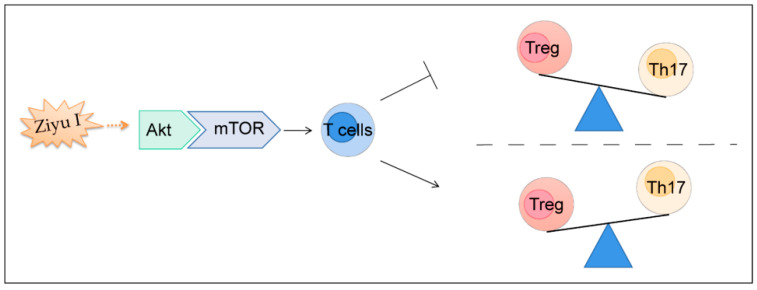
Graphical abstract. The therapeutic effect of Ziyu I on CIA is exerted by inhibiting the activity of Akt-mTOR signaling, reducing the differentiation of Th17, and thereby restoring the balance of Th17/Treg. Ziyu I: Ziyuglycoside I, Akt: protein kinase B 1, mTOR: mechanistic target of rapamycin, Treg: regulatory T cell, Th17: helper T cell 17.

In summary, Ziyu I inhibits the Akt/mTOR signaling pathway in the pathological process of CIA to prevent Th17 cell differentiation and thus restore Th17/Treg balance, alleviating inflammatory arthritic response (Figure 7). The results suggest that Ziyu I may be an active ingredient of Chinese herbal medicine with anti-rheumatism properties, and this study will help propel the development of Ziyu I as a promising prodrug for RA treatment.

## Figures and Tables

**Table 1:** The sequences of RT-qPCR primers.

Genes	Forward Sequence	Reverse Sequence
RORγt	5′-CCGCTGAGAGGGCTTCAC-3′	5′-TGCAGGAGTAGGCCACATTACA-3′
Foxp3	5′-ATGTTCGCCTACTTCAGAA-3′	5′-TCATCTACGGTCCACACT-3′
GAPDH	5′-AAATGGTGAAGGTCGGTGTGAAC-3′	5′-CGACATACTCAGCACCAGCACACT-3′

## Data Availability

The data that support the findings of this study are available from the corresponding author upon reasonable request.
